# Fibrosis-Net: A Tailored Deep Convolutional Neural Network Design for Prediction of Pulmonary Fibrosis Progression From Chest CT Images

**DOI:** 10.3389/frai.2021.764047

**Published:** 2021-11-03

**Authors:** Alexander Wong, Jack Lu, Adam Dorfman, Paul McInnis, Mahmoud Famouri, Daniel Manary, James Ren Hou Lee, Michael Lynch

**Affiliations:** ^1^ Vision and Image Processing Research Group, University of Waterloo, Waterloo, ON, Canada; ^2^ Waterloo Artificial Intelligence Institute, University of Waterloo, Waterloo, ON, Canada; ^3^ DarwinAI Corp., Waterloo, ON, Canada

**Keywords:** pulmonary fibrosis, neural network, computed tomography, chest CT, convolutional, open source

## Abstract

Pulmonary fibrosis is a devastating chronic lung disease that causes irreparable lung tissue scarring and damage, resulting in progressive loss in lung capacity and has no known cure. A critical step in the treatment and management of pulmonary fibrosis is the assessment of lung function decline, with computed tomography (CT) imaging being a particularly effective method for determining the extent of lung damage caused by pulmonary fibrosis. Motivated by this, we introduce Fibrosis-Net, a deep convolutional neural network design tailored for the prediction of pulmonary fibrosis progression from chest CT images. More specifically, machine-driven design exploration was leveraged to determine a strong architectural design for CT lung analysis, upon which we build a customized network design tailored for predicting forced vital capacity (FVC) based on a patient’s CT scan, initial spirometry measurement, and clinical metadata. Finally, we leverage an explainability-driven performance validation strategy to study the decision-making behavior of Fibrosis-Net as to verify that predictions are based on relevant visual indicators in CT images. Experiments using a patient cohort from the OSIC Pulmonary Fibrosis Progression Challenge showed that the proposed Fibrosis-Net is able to achieve a significantly higher modified Laplace Log Likelihood score than the winning solutions on the challenge. Furthermore, explainability-driven performance validation demonstrated that the proposed Fibrosis-Net exhibits correct decision-making behavior by leveraging clinically-relevant visual indicators in CT images when making predictions on pulmonary fibrosis progress. Fibrosis-Net is able to achieve a significantly higher modified Laplace Log Likelihood score than the winning solutions on the OSIC Pulmonary Fibrosis Progression Challenge, and has been shown to exhibit correct decision-making behavior when making predictions. Fibrosis-Net is available to the general public in an open-source and open access manner as part of the OpenMedAI initiative. While Fibrosis-Net is not yet a production-ready clinical assessment solution, we hope that its release will encourage researchers, clinicians, and citizen data scientists alike to leverage and build upon it.

## 1 Introduction

Pulmonary fibrosis is a serious chronic lung disease in which permanent tissue scarring and damage occurs in the lungs. The increasing replacement of healthy lung tissues with fibrotic tissue results in progressive, irreversible reduction in lung function over time. There are currently no known cure and limited treatment options for pulmonary fibrosis. The treatment and management of the disease is currently focused on the attenuation of lung function decline progression and improving quality of life. The rate of progression for a patient with pulmonary fibrosis is highly variable, ranging from little to no change over many years to rapid deterioration in a short period of time.

A very critical step in the treatment and management of pulmonary fibrosis is the assessment of lung function decline. This assessment guides clinicians to determining the best course of treatment and management ranging from oxygen therapy and pulmonary rehabilitation to pharmacological agents [pirfenidone (Taniguchi et al., 2010) and nintedanib (Richeldi et al., 2014)] and lung transplantation (Kistler et al., 2014). Guidelines set out by the ATS/ERS/JRS/ALAT (Raghu et al., 2018) describe a number of methods for diagnosis of pulmonary fibrosis. Invasive techniques, such as surgical lung biopsy, have associated risks to the health and lung function for the patient (Richeldi et al., 2017). Transbroncial lung biopsy is a less invasive technique where small samples of the lung tissue are taken using video-assisted thoracoscopy or flexible bronchoscopy (Tomassetti et al., 2016). A number of methods have been utilized by clinicians for assessing lung function decline after diagnosis. For example, spirometry tests are frequently leveraged for measuring the FVC of the lung which is a key indicator of lung function (Watters et al., 1985; Du Bois et al., 2011; Russell et al., 2016; Wuyts et al., 2016). However, spirometry tests give very limited understanding of the underlying disease mechanisms and disease progression within the lungs, thus making the use of spirometry very limited as a predictor for pulmonary fibrosis disease progression.

One of the most effective methods for assessing lung function decline and the extent of lung damage due to pulmonary fibrosis is computed tomography (CT) imaging, and has become clinically routine to conduct CT imaging in an infrequent manner to get a clearer sense of the underlying disease mechanisms and disease progression within the lungs. Several visual signs in CT scans have been identified and leveraged by radiologists to assess lung function decline from pulmonary fibrosis. The most common visual indicator of pulmonary fibrosis is honeycombing, which present as cystic spaces with irregularly thickened fibrotic tissue walls (Devaraj, 2014). However, given the rate of progression of different patients can be highly variable, the ability to accurately predict the progress of pulmonary fibrosis remains a major challenge. This is further compounded by the fact that some common visual indicators such as honeycombing may not be present at certain stages of progression or even at all (Gruden, 2016). In addition, other atypical patterns mimicking other diseases may be present in the CT scans of patients with pulmonary fibrosis instead [e.g., predominance of ground-glass opacity, consolidation, nodules, and atypical distribution of lesions (Souza et al., 2005), as well as the presence of ground-glass attenuation (Lynch, 1996)]. As such, new methods for improving prediction accuracy when leveraging CT images as a tool for assessing and predicting lung function decline due to pulmonary fibrosis in the future is highly desired.

Motivated by the potential of machine learning for computer-aided clinical decision support for pulmonary fibrosis, in this study we introduce Fibrosis-Net, a deep convolutional neural network design tailored specifically for the prediction of pulmonary fibrosis progression from chest CT images. More specifically, machine-driven design exploration was leveraged to determine a strong architectural design for CT lung analysis. It is upon this architectural design that we build a customized network design tailored for predicting forced vital capacity (FVC) based on a patient’s CT scan, initial spirometry measurement, and clinical metadata. Furthermore, to explore the decision-making behavior of Fibrosis-Net, we leverage an explainability-driven performance validation strategy to audit Fibrosis-Net to verify that predictions are based on relevant visual indicators in CT images. Fibrosis-Net is available to the general public in an open-source and open access manner^1^ as part of the OpenMedAI initiative, an open source initiative for medical artificial intelligence solutions that currently include the COVID-Net (Gunraj and Wong, 2020; Wang et al., 2020; Wong et al., 2021b; Ebadi et al., 2021; Gunraj et al., 2021) initiative, Cancer-Net (Lee et al., 2020) initiative, and the TB-Net initiative (Wong et al., 2021a). While Fibrosis-Net is not yet a production-ready screening solution, we hope that its open source release will encourage researchers, clinicians, and citizen data scientists alike to leverage and build upon them. The application scenario is to leverage infrequent CT imaging acquisition and combine with patient spirometry measurement as well as clinical metadata to predict the FVC at a desired time-point in the future to aid clinicians with treatment and care planning, and is complementary to continuous monitoring through spirometry tests.

The paper is organized as follows. Related work in the area of artificial intelligence for computer-aided clinical decision support for pulmonary fibrosis is discussed in Related Work section. The Materials and Methods section provides a detailed description of the data preparation and analysis process, the architecture design construction process, the proposed Fibrosis-Net network architecture design, and the explainability-driven performance validation process. The Results section presents both the quantitative performance validation results evaluating the efficacy of the proposed Fibrosis-Net using a patient cohort from the OSIC Pulmonary Fibrosis Progression Challenge (OSIC, 2020) as well as visual validation results from the explainability-driven performance validation process used to study the decision-making behavior of Fibrosis-Net. Finally, conclusions are drawn and future work is discussed in the Conclusions Section, where the broader impact of the proposed work is also discussed.

## 2 Related Work

Motivated by the significant benefits that can be gained and the challenges involved from a clinical perspective, there has been a recent interest in leveraging artificial intelligence for computer-aided clinical decision support of pulmonary fibrosis based on CT images (Levin, 2018; Walsh et al., 2018; Christe et al., 2019; Walsh et al., 2020). For instance, Anthimopoulos et al. (2016) use a deep convolutional neural network to analyze 2D patches from the CT image to classify reticulation, honeycombing, ground glass opacity (GGO), consolidation, and micronodules in lung tissue. Christodoulidis et al. (2016) proposed a multi-source transfer learning approach with deep convolutional neural networks pre-trained with a selection of texture data sets, again with the goal of classifying 2D image patches of lung tissue in CT. Bermejo-Peláez et al. (2020) describe a method using an ensemble of deep convolutional neural networks where the output of each network is summed up and weighted before being combined to form the overall output of the ensemble.

More recently, the significant potential and need for advancements in artificial intelligence-driven methods for computer-aided clinical decision support of pulmonary fibrosis was exemplified by the Kaggle Pulmonary Fibrosis Progression Challenge (OSIC, 2020). This challenge was launched by the Open Source Imaging Consortium (OSIC) to get the research community to accelerate advancement of machine learning for pulmonary fibrosis assessment. To the best of the authors’ knowledge, the patient cohort curated by OSIC for the challenge is the largest publicly available cohort in literature. Amongst the many artificial intelligence solutions introduced as part of the challenge, the 1st place winning solution (OSIC, 2020) proposed a weighted ensemble between a deep convolutional neural network with a state-of-the-art EfficientNet-B5 network architecture design (Tan and Le, 2020) and a multiple quantile regressor to predict the lung function decline of a patient based on a patient’s CT scans, initial spirometry measurement, and clinical metadata.

To the best of the authors’ knowledge, the notion of explainability-driven performance validation of the decision-making behavior of artificial intelligence solutions for the prediction of pulmonary fibrosis progression have not been previously explored in literature. Such validation can be very valuable for driving greater clinical adoption of such solutions in a transparent and trusted manner. Furthermore, while explainability-driven performance validation strategies has been demonstrated to be very successful in past studies for the purpose of clinical classification (Gunraj and Wong, 2020; Wang et al., 2020; Wong et al., 2021a; Gunraj et al., 2021), to the best of the authors’ knowledge this is the first study in literature to successfully leverage explainability-driven performance validation on a clinical regression problem.

## 3 Materials and Methods

### 3.1 Data Preparation and Analysis

To build the proposed Fibrosis-Net, we leverage the patient cohort from the OSIC Pulmonary Fibrosis Progression Challenge (OSIC, 2020). The data for this patient cohort consists of chest CT scans, forced vital capacity (FVC) measurements from frequent visits over the course of around 1–3 years, and associated clinical metadata (i.e., age, sex, smoking status, and patient’s relative FVC measurement compared to the typical FVC measurement of a patient with similar characteristics). More specifically, in this study, the training set consists of 172 patient cases, while the test set consists of approximately 28 patient cases. To the best of the authors’ knowledge, the patient cohort curated for this challenge is the largest publicly available cohort in literature.

Table 1 summarizes the demographic variables of the data from the patient cohort in the OSIC Pulmonary Fibrosis Progression Challenge (OSIC, 2020) used in this study. A number of observations can be made based on the demographic distribution analysis. First, it can be observed that the patient cases in the cohort are distributed across the different age groups, with the mean age being 67.1 and the highest number of patients in the cohort are between the ages of 60–69. This distributional trend towards older adults is reflective of the fact that pulmonary fibrosis is typically diagnosed later in life due to the condition worsening as time goes by. Second, it can be observed that the majority of patient cases in the cohort are individuals who have smoked sometime in their life, with the majority of patients being ex-smokers. This distributional trend is reflective of the fact that a recognized risk factor for the development of pulmonary fibrosis is smoking. Furthermore, smoking can lead to more significant detrimental effects that can reduce the survival rate of patients with pulmonary fibrosis. Third, it can be observed that a majority of the patient cases in the cohort are male. This is consistent with clinical studies in literature showing that gender differences in the risk of pulmonary fibrosis (Ekström. et al., 2014) as well as at clinical presentation (Kalafatis et al., 2019). Finally, it can be observed that the patient cases span a wide range of relative FVC measurement values, which indicates different levels of lung function decline. This distribution diversity in relative FVC measurement values is desirable as it allows the deep neural network being trained to be exposed to a wider variety of pulmonary fibrosis progression scenarios for greater generalizability for different patient conditions.

**TABLE 1:** Summary of demographic variables of data used in this study.

Age	Mean ± std	67.14 ± 7.01 (%)
	50–59	14.9
	60–69	47.9
	70–79	34.4
	80–89	2.8
**Sex**		
	Male	78.55
	Female	21.45
**Smoking status**		
	Currently smokes	5.4
	Ex-smoker	66.3
	Never smoked	28.3
**Relative FVC**		
	< 50	3.4
	50–69	37.0
	70–89	36.8
	90–109	15.2
	110+	7.6

### 3.2 Data Processing

**FIGURE 1:**
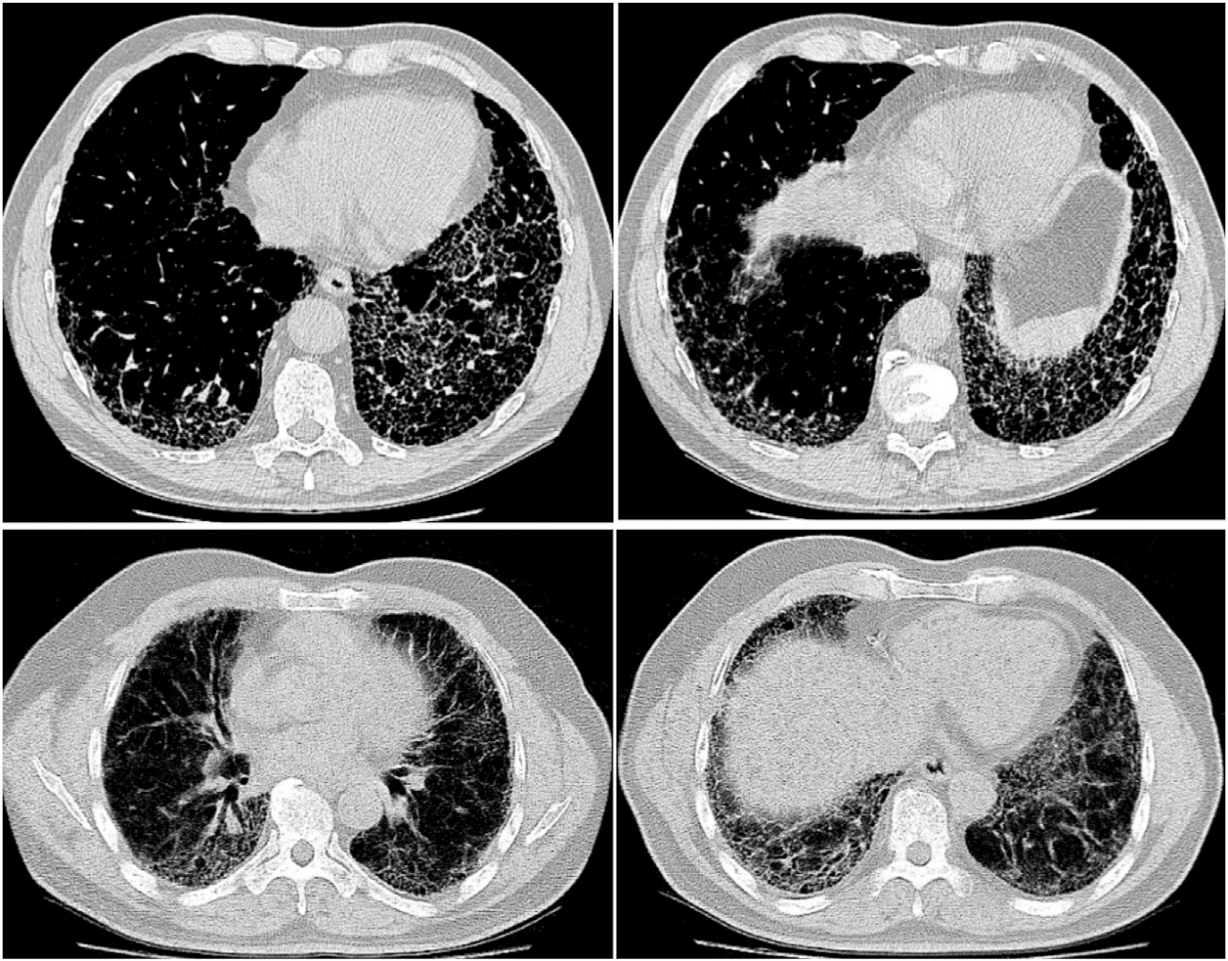
Example CT slices from the patient cohort from the OSIC Pulmonary Fibrosis Progression Challenge (OSIC, 2020).

The data processing pipeline used in this study is as follows. A number of pre-processing steps were conducted to improve the consistency and quality of the CT images from the patient scans. More specifically, all CT imaging data was translated to Hounsfield units (HU), and windowing was performed with a window level of -650 HU and a window width of 1700 HU to better focus on clinically relevant lung features. Furthermore, synthetic padding and circular artifacts found in the CT imaging data for several patient cases within the patient cohort are mitigated to reduce the likelihood of erroneous visual features from being learned as predictive indicators. Finally, calibration value errors found in the data for several patient cases within the patient cohort are accounted for to further mitigate the likelihood of erroneous characteristics from being learned as predictive indicators. Example CT slices from the patient cohort are shown in Figure 1. It can be observed that the visual appearance of pulmonary fibrosis in different patient CT scans can be quite varied, and thus can be quite challenging to utilize for lung function decline prediction. The variable visual appearance in CT scans further motivate the exploration of deep learning strategies for tackling such a complex prediction task to facilitate for computer-assisted clinical decision support.

### 3.3 Machine-Driven Design Exploration

The goal of the proposed Fibrosis-Net is to predict the forced vital capacity (FVC) of a patient (in ml) at a specific time-point in the future given a patient’s CT scan, initial spirometry measurement, and clinical metadata. In order to construct a highly customized deep convolutional neural network architecture design tailored specifically for high predictive performance, we take inspiration from (Wang et al., 2020) and a generative synthesis (Wong et al., 2018) approach was leveraged as the machine-driven design exploration strategy for determining a strong architectural design for CT lung analysis. In this approach, the problem of determining a tailored deep neural network architecture design is formulated as a constrained optimization problem based on a universal performance function u [e.g., (Wong, 2018)] and a set of quantitative constraints. The aforementioned constrained optimization problem is solved in an iterative fashion, based on an initial network design prototype, the set of quantitative constraints, and data at hand, to produce network *N*.

More specifically, the backbone architecture design for CT lung analysis identified via machine-driven design exploration leveraged residual architecture design principles (He et al., 2016a; He et al., 2016b) as an initial network design prototype. In addition, the machine-driven design exploration leveraged 2,116 patient cases acquired from around the world both with presence and absence of respiratory diseases for improve the quantity and diversity of CT scans, along with associated predictive performance constraints (Gunraj and Wong, 2020; Gunraj et al., 2021). It is upon this backbone architecture design that the proposed Fibrosis-Net network architecture design was built to be tailored specifically for predicting FVC based on the CT scan, initial spirometry measurement, and clinical metadata of a patient.

### 3.4 Network Architecture

**FIGURE 2:**
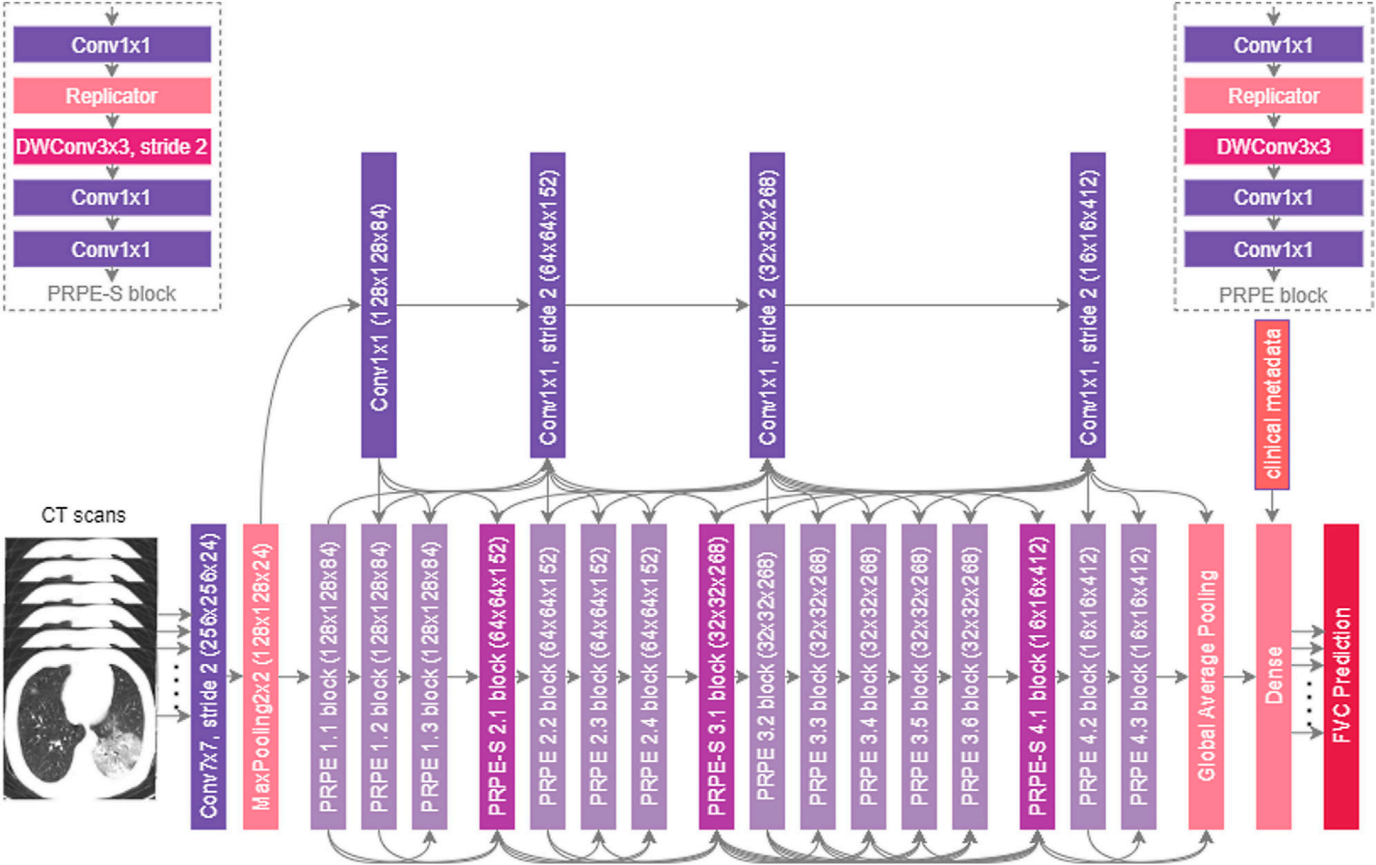
The proposed Fibrosis-Net architecture design. Given a patient’s CT scan, initial spirometry measurement, and clinical metadata, the proposed Fibrosis-Net predicts the forced vital capacity (FVC) of a patient at a specified time-point in the future. Fibrosis-Net exhibits an efficient network architecture design with light-weight components that strikes a strong balance between representational capacity and architecture and computational efficiency.

The proposed Fibrosis-Net architecture is shown in Figure 2. Given a stack of CT images from a patient’s CT scan, each CT image from the lower 55% subset of the CT scan (where pulmonary fibrosis typically presents itself in the lungs) is passed through a series of convolutional layers to create a condensed feature representation characterizing the CT image. This condensed feature representation of the CT image, along with clinical metadata, are then passed together into a dense layer to predict the linear rate of change in lung function. The predicted linear rates of change in lung function from all of the CT images in a patient’s CT scan are then passed into the FVC prediction layer, where the median predicted linear rate of change in lung function, the initial spirometry measurement of the patient, and clinical metadata (i.e., age, sex, and smoking status) are leveraged to predict the FVC at the desired time-point. As the final operations in the FVC prediction layer, a regressor fitted on the clinical metadata with Elastic Net regularization is also leveraged to produce a predicted FVC at the desired time-point, which is then combined with the convolutional-driven FVC prediction to obtain the final FVC prediction.

#### 3.4.1 Architectural Heterogeneity and Efficiency

The proposed Fibrosis-Net possesses a highly efficient, heterogeneous design comprising largely of lightweight architectural components such as depthwise convolutions and pointwise convolutions. In particular, similar to (Gunraj and Wong, 2020; Gunraj et al., 2021), both unstrided and strided projection-replication-projection-expansion design patterns (denoted as PRPE and PRPE-S for unstrided and strided patterns, respectively) are heavily utilized to strike a strong balance between representational capacity and architectural and computational efficiency. The efficiency of the proposed Fibrosis-Net makes it very well-suited for clinical scenarios where computational resources are limited, particularly when dealing with CT scans consisting of many CT images such as the pulmonary fibrosis progression prediction task in this study.

#### 3.4.2 Selective Long-Range Connectivity

Furthermore, selective long-range connectivity is exhibited in the proposed Fibrosis-Net, with central connectivity hubs comprised of pointwise convolutions for enabling flow in information directly from earlier convolutional layers to the later convolutional layers. By keeping the quantity of long-range connections very sparse through centralized connectivity hubs, the selective long-range connectivity characteristics of Fibrosis-Net strikes a strong balance between improved representational capacity and architectural efficiency.

#### 3.4.3 Visual-Clinical Feature Fusion

It can also be observed that Fibrosis-Net fuses learned visual features from a patient’s CT scan with clinical metadata at a later stage of the network architecture design. This enables the efficient utilization of important clinical knowledge captured within clinical metadata alongside important visual characteristics captured in a patient’s CT scan for more well-informed, comprehensive predictive capabilities. Finally, the utilization of a flexible FVC prediction layer in the proposed Fibrosis-Net architecture design takes into account a variable set of linear rates of change in lung function predicted by the dense layer depending on the quantity of CT images within a particular patient’s CT scan. As a result, this effectively allows for much greater flexibility in dealing with different real-world scenarios where the imaging protocol and imaging equipment parameters and configurations can vary greatly.

These architectural characteristics possessed by the proposed Fibrosis-Net illustrates the effectiveness of leveraging machine-driven design exploration for constructing customized deep neural network architecture designs that are specifically tailored for clinical decision support tasks.

### 3.5 Implementation, Training, and Evaluation of Fibrosis-Net

The resulting Fibrosis-Net was implemented using the TensorFlow deep learning library (Abadi et al., 2015). Training of Fibrosis-Net was conducted with an Intel Core i9-9820X CPU and an Nvidia GeForce RTX 2080 Ti GPU using the Adam optimization algorithm (Kingma and Ba, 2017). The loss function used was a mean absolute error (MAE) loss, with a learning rate of 1e-4, exponential decay of 0.99 every 100 steps, and a batch size of 8. Evaluation of Fibrosis-Net from an inference perspective was conducted on an Intel Core i7-8750H CPU.

### 3.6 Explainability-Driven Performance Validation of Fibrosis-Net

Understanding the behavior of a deep neural network when making predictions is very important when used in clinical decision support scenarios, given that such predictions will impact patient care and influence treatment and management planning. Inspired by this, we leverage an explainability-driven performance validation strategy to study the decision-making behavior of Fibrosis-Net as to verify that predictions are based on relevant visual indicators in CT images. Similar to (Gunraj and Wong, 2020; Wang et al., 2020; Wong et al., 2021a; Gunraj et al., 2021), we leverage GSInquire (Lin et al., 2019) as the explainability method of choice for explainability-driven performance validation in this study to identify critical visual factors in CT images that Fibrosis-Net leverages to make FVC predictions. More specifically, GSInquire harnesses the same generative synthesis strategy (Wong et al., 2018) leveraged in the machine-driven design exploration process, as a previous study demonstrated the ability of GSInquire to provide explanations that better reflect the decision-making process of deep neural networks quantitatively when compared to other state-of-the-art explainability methods (Lin et al., 2019). More importantly, GSInquire is, to the best of the authors’ knowledge, one of the only explainability methods in literature that can be leveraged for studying and validating clinical regression problems, and thus makes it highly desirable for this study.

In brief, GSInquire utilizes the generative synthesis (Wong et al., 2018) approach from the machine-driven design exploration process for identifying the backbone architecture design, where an inquisitor ℐ is leveraged to probe a network *N* with input *x*. The reactionary responses *y* from the probing process are leveraged by ℐ to produce quantitative interpretation *z* of the decision-making process of a deep neural network *N* given an input *x* in the same sub-space as *x*. The details pertaining to GSInquire for explaining the decision-making behavior of deep neural networks on CXR images can be found in (Wang et al., 2020). An interesting property of GSInquire that also makes it well-suited for explainability-driven performance validation is that it is capable of producing explanations identifying specific critical factors within an image that quantitatively impacts the decisions made by a deep neural network, thus making it more readily interpretable and more quantitative for validation purposes than the types of relative importance variations visualized by other methods.

The details regarding how GSInquire can be leveraged to produce interpretations of deep neural network decision-making behavior for clinical prediction tasks can be found in (Wang et al., 2020). Here, the interpretation *z* indicates the critical visual factors leveraged by Fibrosis-Net from CT images when making FVC predictions.

## 4 Results

To explore and evaluate the efficacy of the proposed Fibrosis-Net for the prediction of lung decline progression due to pulmonary fibrosis from chest CT images, we take a multi-prone approach where we conduct: 1) an empirical quantitative performance evaluation of the deep neural network design to study its performance compared to last state-of-the-art methods, as well as 2) a visual validation evaluation of the decision-making behavior of the deep neural network design using an explainability-driven performance validation process. The quantitative and qualitative results are presented and discussed in detail below.

### 4.1 Quantitative Performance Validation Results

We quantitatively evaluate the efficacy of the proposed Fibrosis-Net using the OSIC Pulmonary Fibrosis Progression Challenge test cohort (OSIC, 2020). As consistent with the evaluation procedure described in (OSIC, 2020), the modified Laplace Log Likelihood score is used, and a comparative analysis is conducted against the three Kaggle winning methods in the OSIC Pulmonary Fibrosis Progression Challenge. The modified Laplace Log Likelihood score (denoted here as *L*) can be expressed as,

$$\sigma_{\mathrm{clipped}}=max\left(\sigma ,70ml\right),$$ (1)

$$\Delta =min\left(\left|\mathrm{FV}C_{\mathrm{true}}-\mathrm{FV}C_{\mathrm{predicted}}\right|,1000ml\right),$$ (2)

$$L=-\frac{\sqrt{2\Delta }}{\sigma_{\mathrm{clipped}}}-\ln\left(\sqrt{2}\sigma_{\mathrm{clipped}}\right),$$ (3)

where Δ is a prediction error that is threshold at 1,000 ml to mitigate adverse penalization, *σ*_*clipped*_ is the confidence value clipped at 70 ml to approximate measurement uncertainty. As such, the modified Laplace Log Likelihood score *L* accounts for both the accuracy of each prediction via *δ* as well as the certainty of each prediction via *σ*_*clipped*_. The modified Laplace Log Likelihood score is negative in value and higher the score is, the better the performance of the method is for predicting pulmonary fibrosis progression.

The modified Laplace Log Likelihood scores for the proposed Fibrosis-Net and the winning methods are shown in Table 2. It can be observed that Fibrosis-Net achieves a significantly higher modified Laplace Log Likelihood score when compared to the winning solutions in the challenge. More specifically, Fibrosis-Net achieved a Laplace Log Likelihood score that exceeded the Kaggle 1st place winning solution by **0.0117**, which is significantly higher than the score gaps between the three winning solutions given the logarithmic scale of the modified Laplace Log Likelihood score. Based on these results, it can be seen that Fibrosis-Net can achieve state-of-the-art performance for lung decline progression and demonstrates the efficacy of machine-driven design exploration for constructing deep neural network designs tailored for clinical decision support tasks.

**TABLE 2:** Comparison of Laplace Log Likelihood scores for the Kaggle winning methods and the proposed Fibrosis-Net on the test cohort from the OSIC Pulmonary Fibrosis Progression Challenge.

Method	Laplace log likelihood
Kaggle 1st place (OSIC, 2020)	−6.8305
Kaggle 2nd place (OSIC, 2020)	−6.8311
Kaggle 3rd place (OSIC, 2020)	−6.8336
Fibrosis-Net	−**6.8188**

Notes: Best results highlighted in bold.

Next, we evaluate the efficiency of the proposed Fibrosis-Net and its suitability for clinical scenarios where computational resources are limited. More specifically, we computed the architecture complexity and computational efficiency of the backbone architecture of the proposed Fibrosis-Net as well as that of the Kaggle 1st place winning solution in terms of number of parameters and inference speed. The architectural complexity and computational efficiency of the proposed Fibrosis-Net and the Kaggle 1st place winning solution are shown in Table 3. It can be observed that the backbone architecture of the proposed Fibrosis-Net has >22× lower architectural complexity >10× higher computational efficiency than that of the Kaggle 1st place winning solution. As such, the significantly lower architectural complexity and computational complexity of the proposed Fibrosis-Net makes it well suited for clinical scenarios with limited computational resources.

**TABLE 3:** Comparison of architectural complexity (number of parameters) and computational efficiency (inference speed) for the Kaggle 1st place winning method and the proposed Fibrosis-Net on the test cohort from the OSIC Pulmonary Fibrosis Progression Challenge.

Method	Number of parameters (millions)	Inference speed (s)
Kaggle 1st place (OSIC, 2020)	30.6	0.55
Fibrosis-Net	**1.38**	**0.053**

Notes: Best results highlighted in bold.

### 4.2 Explainability-Driven Performance Validation Results

**FIGURE 3:**
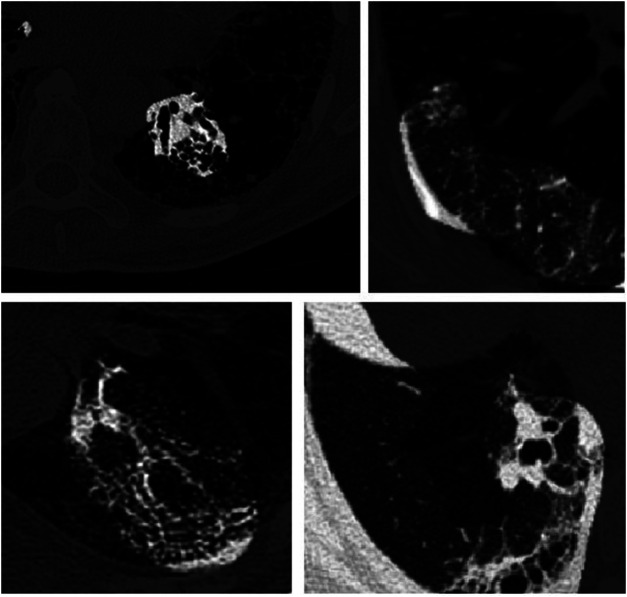
Example critical factors (highlighted as brighter regions) in example CT images of pulmonary fibrosis patients as identified by GSInquire (Lin et al., 2019). It can be observed that Fibrosis-Net is capable of leveraging clinically relevant visual indicators such as the presence and geographic extent of honeycombing in the lungs as presented in the CT images to make FVC predictions.

As discussed earlier, we harnessed GSInquire (Lin et al., 2019) to conduct explainability-driven performance validation of Fibrosis-Net in order to study its behavior when making predictions of lung function decline, as well as validate whether predictions are based on clinically-relevant imaging features rather than based on irrelevant features. Figure 3 illustrates example critical factors in CT images of pulmonary fibrosis patients as identified by GSInquire that are key to the decision-making behavior of the proposed Fibrosis-Net. It can be observed that Fibrosis-Net is capable of leveraging clinically relevant visual indicators such as the presence and geographic extent of honeycombing in the lungs as presented in the CT images to make FVC predictions. As such, it can be clearly seen that the proposed Fibrosis-Net is driven by correct, clinically relevant decision-making behavior when making predictions of pulmonary fibrosis progression similar to those leveraged by clinicians (Devaraj, 2014). These visual results also highlight the importance of harnessing explainability-driven performance validation when building and evaluating deep neural networks for clinical decision support tasks.

## 5 Discussion

There are several important benefits to taking such an explainability-driven approach to performance validation, particularly for the proposed Fibrosis-Net where the purpose relates to clinical decision support. First of all, by leveraging explainability-driven performance validation, one can obtain greater transparency and understanding into the decision-making behavior of a deep neural network to ensure that it is leveraging clinically-relevant imaging features to make decisions (i.e., “making the right decisions for the right reasons“). Second, one can gain much greater insight into potential gaps, biases, and errors in both the data as well as in the decision-making behavior of a deep neural network (i.e., “making the right decisions for the wrong reasons“ based on irrelevant features such as synthetic padding, circular artifacts, etc.). Third, by providing greater transparency into the decision-making processing during prediction, one can provide a greater sense of trust for clinicians leveraging such deep neural networks for computer-assisted clinical decision support and drive greater clinical adoption of such artificial intelligence-driven technologies.

Based on both quantitative and qualitative results, it was demonstrated that Fibrosis-Net can not only make FVC predictions at a higher level of accuracy than state-of-the-art methods, but also do it in a more trustworthy, validated manner that leverages clinically relevant visual indicators within the CT images of a pulmonary fibrosis patient.

Given the devastating effects of pulmonary fibrosis on a individual’s health and well-being and the lack of a known cure, the research in lung function decline prediction presented in this study can have positive benefit to clinical scientists and researchers who are developing deep learning systems for supporting clinical workflows in a number of impactful ways. First, by illustrating the efficacy of machine-driven design for building highly tailored deep neural network architecture designs for a prediction task beyond the types of clinical decision support tasks illustrate in past studies (Gunraj and Wong, 2020; Wang et al., 2020; Wong et al., 2021b; Gunraj et al., 2021), the hope is that other researchers and scientists may consider leveraging such an approach to accelerate and improve the design of deep learning solutions for different clinical scenarios. Second, by illustrating the efficacy of explainability-based performance validation on gaining a better understanding of the behavior of Fibrosis-Net on making FVC predictions, the hope is that other researchers and scientists may consider leveraging explainability methods more frequently to improve transparency and trust. Third and finally, the proposed Fibrosis-Net is released in an open-access, open-source fashion, thus allowing for researchers, scientists, and clinicians to leverage this work for further investigation and build upon this work to accelerate the development of clinically viable systems.

## 6 Conclusion

In this study, we introduced Fibrosis-Net, a deep convolutional neural network design tailored for the prediction of pulmonary fibrosis progression from CT images. Designed with the help of a machine-driven design exploration strategy, Fibrosis-Net is available open source to the general public as part of the OpenMedAI initiative. Experimental results using a patient cohort from the OSIC Pulmonary Fibrosis Progression Challenge show that the proposed Fibrosis-Net can achieve state-of-the-art forced vital capacity prediction performance when compared to the winning solutions on the challenge. Furthermore, an explainability-driven performance validation of Fibrosis-Net showed that relevant visual indicators in the CT images were leveraged when producing predictions. Given the promise of the proposed Fibrosis-Net, we aim to explore this strategy for creating deep neural networks to perform other clinical decision support tasks for other pulmonary conditions such as chronic obstructive pulmonary disease prediction and pulmonary hypertension detection.

It is important to note that Fibrosis-Net is by no means a production-ready clinical assessment solution, and is intended as a foundation for further research and development. Furthermore, it is important to note that the predictions made by Fibrosis-Net and other similar artificial intelligence clinical decision support tools should not be accepted blindly but rather by utilized to aid clinicians in the clinical decision support process. As such, much greater impact can be achieved with tools such as Fibrosis-Net are utilized in a human-in-the-loop manner. As future work, the aim is to conduct a deeper exploration and analysis using explainability methods such as GSInquire into a larger corpus of patient cases to get a deeper understanding of disease mechanisms to garner new clinical and model insights Carlson et al., 2017, Nalysnyk et al., 2020.

## Data Availability

Publicly available datasets were analyzed in this study. This data can be found here: https://www.kaggle.com/c/osic-pulmonary-fibrosis-progression.

## Footnotes

1
https://github.com/darwinai/FibrosisNet.
